# Computational psychopharmacology: a translational and pragmatic approach

**DOI:** 10.1007/s00213-019-05302-3

**Published:** 2019-07-04

**Authors:** Trevor W. Robbins, Rudolf N. Cardinal

**Affiliations:** 1 Department of Psychology, University of Cambridge, Downing Street, Cambridge, CB23EB UK; 2 Behavioural and Clinical Neuroscience Institute, University of Cambridge, Cambridge, UK; 3 Department of Psychiatry, University of Cambridge, Cambridge, UK; 4 Liaison Psychiatry Service, Cambridgeshire & Peterborough NHS Foundation Trust, Cambridge, UK

**Keywords:** Computer modelling, Reinforcement learning, Dopamine, Serotonin, Depression, Schizophrenia

## Abstract

**Rationale:**

Psychopharmacology needs novel quantitative measures and theoretical approaches based on computational modelling that can be used to help translate behavioural findings from experimental animals to humans, including patients with neuropsychiatric disorders.

**Objectives:**

This brief review exemplifies this approach when applied to recent published studies of the effects of manipulating central dopaminergic and serotoninergic systems in rodents and marmoset monkeys, and possible comparisons with healthy human volunteers receiving systemic agents or patients with depression and schizophrenia.

**Methods:**

Behavioural effects of central depletions of dopamine or serotonin in monkeys in probabilistic learning paradigms are characterised further by computational modelling methods and related to rodent and human data.

**Results:**

Several examples are provided of the power of computational modelling to derive new measures and reappraise conventional explanations of regional neurotransmitter depletion and other drug effects, whilst enhancing construct validation in patient groups. Specifically, effects are shown on such parameters as ‘stimulus stickiness’ and ‘side stickiness’, which occur over and above effects on standard parameters of reinforcement learning, reminiscent of some early innovations in data analysis in psychopharmacology.

**Conclusions:**

Computational modelling provides a useful methodology for further detailed analysis of behavioural mechanisms that are affected by pharmacological manipulations across species and will aid the translation of experimental findings to understand the therapeutic effects of medications in neuropsychiatric disorders, as well as facilitating future drug discovery.

Psychopharmacology is a quantitative branch of neuroscience that relates behavioural effects of drugs in the laboratory or the clinical context to their underlying mechanisms. From the pharmacological perspective, the ability of the investigator to vary the dose of the active drug so as to produce graded behavioural effects provides an exquisite test of the relationship. From the experimental psychologist’s perspective, it is desirable to seek the most sensitive measures for that relationship, which may involve transforming the dependent variables in theoretically coherent ways, using computational methods, to attain insight into controlling processes and mechanisms. An excellent example is the use of signal detection theory (Green and Swets 1966) which can dissect discrimination performance into two orthogonal factors—discriminative sensitivity (measured by d’) and response bias (β or c). The latter provides an overall tendency to make a particular choice (e.g. to respond or not, to answer ‘yes’ or ‘no’) which could arise from response strategy or motivational factors. Warburton and Brown (1972) introduced such methods into psychopharmacology to show that cholinergic agents such as physotigmine, at least at some doses in rats, improved d’ with no effects on β, suggesting that the drug enhanced visual attention.

Bernard Weiss and Victor Laties (Weiss and Gott 1972; Laties 1972) also pioneered the use of sophisticated variables to characterise drug effects and several investigators have employed microanalyses of behaviour to look for understanding at the level of ongoing sequential changes in behaviour. For example, Koek and Slangen (1984), using a measure of the conditional tendency to repeat responding [p(rep)], found that amphetamine had a tendency to increase response repetition that overshadowed its effects on discrimination per se. Sahgal and Clincke (1985) pointed out potential problems with the p(rep) measure and introduced more refined indices that were shown to be sensitive to this tendency under amphetamine.

Although there is much to be said for examining effects of drugs on stable baselines of choice behaviour (established, for example, by training) in order to analyse processes such as attention, it is also important to quantify effects of drugs on the dynamic processes of learning—although it is more difficult and costly to perform adequate dose-response analyses. Traditionally, learning can be measured crudely in terms of the number of trials, or errors made, that it takes to reach a suitable criterion of discrimination. However, reinforcement learning theory has provided us with a number of algorithms that adequately support learning, ranging from agent-based approaches (Russell and Norvig 1995; Sutton and Barto 1998) to more psychologically motivated models such as that of Rescorla and Wagner (1972) for Pavlovian conditioning. Learning can be interpreted as a hypothesis testing or environmental modelling process by which the animal (or human) predicts outcomes of its behavioural choices. Mechanistically, learning can be driven by ‘prediction errors’ that signal a difference between expected outcomes and obtained outcomes, updating pre-existing evidence of the associative strength of a specific response with this new evidence. Prediction errors can thus be seen as part of the brain’s Bayesian updating process. Modern computing has also brought the ability to apply formal Bayesian computational approaches analytically, providing a rich potential basis for analysing drug effects on learning. This has become an exciting endeavour, given that we not only understand much about how drugs such as amphetamine affect neurotransmitter function, but also because of discoveries that neurotransmitters such as dopamine (DA) and serotonin (5-hydroxytryptamine, 5-HT) play important roles in learning as well as performance. Thus, Schultz et al. (1997), based on such previously hypothesised mechanisms (Montague et al. 1996), were able to show in rhesus monkeys that the phasic firing of mid-brain DA neurons in response to conditioned stimuli (CSs) associated with reward provided the neuronal substrate of prediction errors. These neurons’ behaviour conformed to classical learning algorithms, including the Rescorla-Wagner equation, where learning is essentially represented as:

$$ \Delta {V}_{\mathrm{CS}}=\kern0.75em \alpha \beta \left(\lambda \hbox{--} {V}_{\mathrm{total}}\right) $$

where Δ*V*_CS_ represents the change in learned association for a CS, (*λ* – *V*_total_) represents a mismatch (prediction error) between expected (*V*_total_) and obtained (*λ*) outcomes, and *α* and *β* are constants for the CS (predictor) and the unconditioned stimulus (US or outcome), respectively. In the original statement of this learning theory (Rescorla and Wagner 1972, p. 75–76), *λ* is described as the asymptote of learning on a given trial, which depends on the US presented and may be 0 for nonreinforcement. The parameter *α* is a learning rate associated with the CS (to capture the concept that different CSs may have different salience and thus support learning at different rates) and *β* is a learning rate associated with the US (to support the assumption that different USs might similarly support learning at different rates); *α* and *β* are constrained to the range [0, 1] (Box 1).

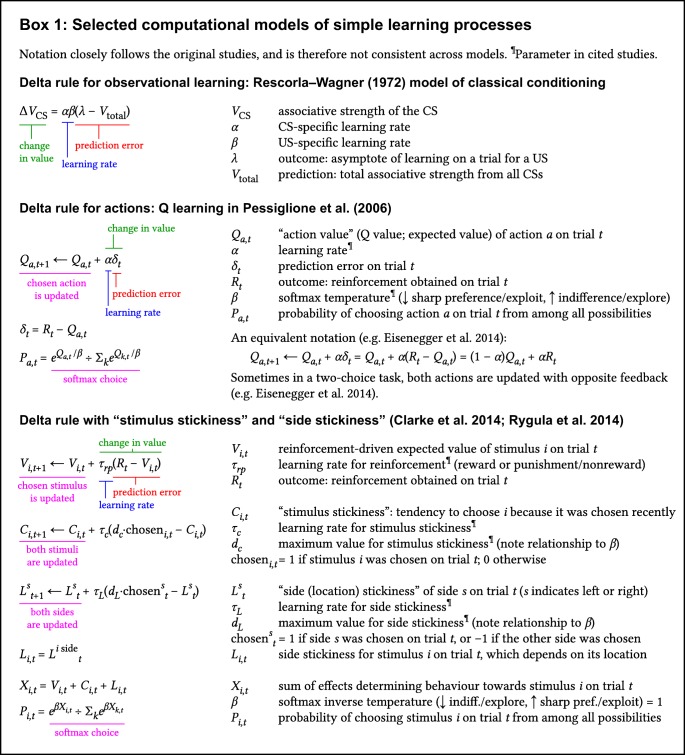



In humans, prediction errors have been confirmed during reinforcement learning via the haemodynamic blood-oxygen-level-dependent functional magnetic resonance imaging (BOLD fMRI) response. Typical paradigms have used probabilistic discrimination learning in which subjects learn to choose by trial and error which of two stimuli is more likely associated with reinforcement when one stimulus on average is rewarded, for example, on 75% of trials and for the other stimulus on 25% of trials, with punishment (or non-reward) occurring on the other trials in reciprocal manner (i.e. 25% and 75% respectively). The initial probabilistic learning phase may be followed by one or more reversals of the contingencies to further assess the flexibility of learning (Fig. 1). Such paradigms have revealed activity during learning and reversal in such structures as the orbitofrontal and ventrolateral prefrontal cortex and the ventral striatum, which are in receipt of dopaminergic afferents (O’Doherty et al. 2001; Cools et al. 2002).

**Fig. 1:**
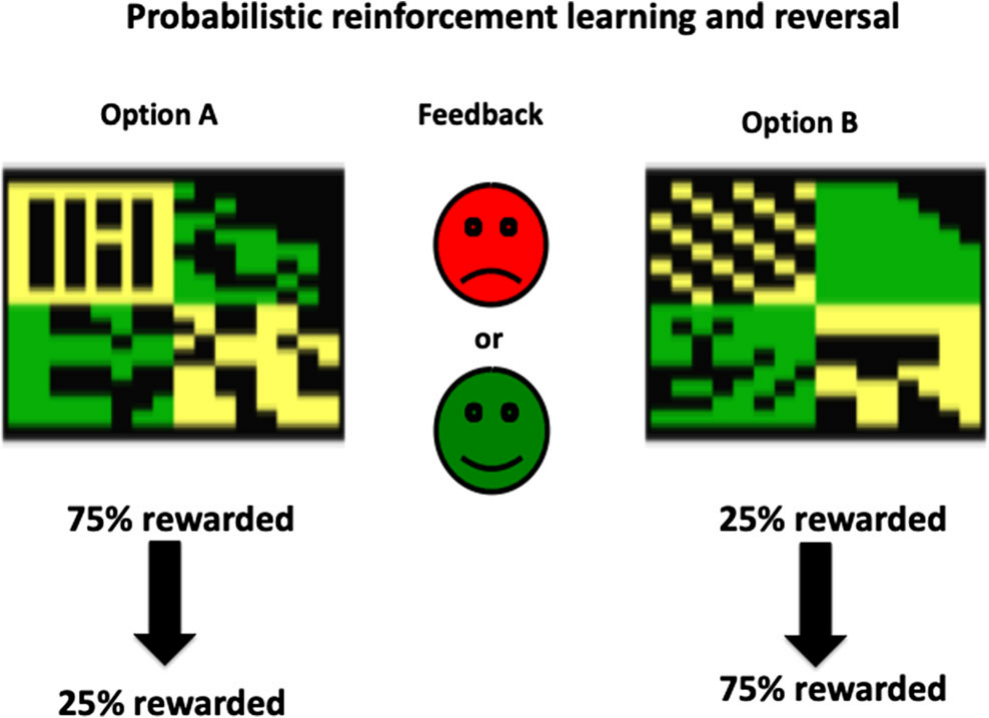
Typical visual stimuli and reinforcement contingencies employed for human studies of probabilistic learning and reversal in discrete trial procedures involving forced choices between option A and option B. Participants are instructed to obtain the most rewards as possible (best achieved here by choosing exclusively the 75% rewarded stimulus). Rewarding outcomes are denoted by brief immediate feedback from the happy face presentation, and punishing outcomes by the frowning face. Following attainment of a learning criterion over a suitable number of trials, the contingencies may be reversed without warning. Such paradigms can be employed to model reinforcement learning in humans and experimental animals. The actual probabilities of reinforcement may vary from study to study. Taken from Cools et al. (2002)

## Reinforcement learning theory and dopamine

It is obviously more difficult in human studies to measure the activity of DA neurons specifically, although it is possible to employ relatively specific DA agonists and antagonists that can be used to test the causal validity of the correlation between DA neuron firing and behaviour. One of the first such studies (Pessiglione et al. 2006) compared the effects of systemic doses of 3,4-dihydroxy-L-phenylalanine (l-dopa) and the D2/3 DA receptor antagonist haloperidol, together with a placebo control, to assess effects of manipulations of DA function in three separate groups of human volunteers performing an instrumental probabilistic discrimination learning paradigm. In this procedure, subjects were rewarded on 75% of occasions for choosing option A, signalled by a distinctive visual fractal stimulus, and only 25% for option B, signalled by a second fractal stimulus (analogous to, but not the same as, to those shown in Fig. 1). In such a task, the optimal strategy for maximising rewards is to choose option A 100% of the time, even though this inevitably results in being punished 25% of the time. Pessiglione et al. also interleaved such discrimination trials with other trials using a different pair of distinctive icons that were associated with avoiding losses instead of gaining rewards. They reported significant differences in reinforcement learning between the two DA manipulations, associated with alterations in BOLD prediction error signals in specific DA terminal regions. Under l-dopa treatment, subjects accrued more rewards during learning than under haloperidol, although there were no effects on losses. By using the striatal BOLD responses to estimate prediction errors, these authors were able to show that the drug effects on behavioural choice conformed to a standard action–value computational model of reinforcement learning (see Fig. 1 of Pessiglione et al. 2006 and Box 1). This included the standard parameters of learning rate (*α*) and ‘temperature’ (*β*), adjusted to maximise the likelihood of the actual choices of the subjects under the model.

This elegant demonstration naturally raises several issues, including the pharmacological specificity of the agents employed, the contribution of individual differences, and the precise functional interpretation of parameters such as *β* (temperature) which could, for example, reflect motivational factors or general strategic tendencies such as ‘win-stay and lose-shift’ or ‘exploration vs exploitation’ (e.g. the overall ratio of stay to shift responses) (Cohen et al. 2007; Frank et al. 2009).

A problem we have observed, especially in human studies, is the difficulty of performing dose-response studies, particularly at low doses of certain agents that may affect presynaptic ‘autoreceptors’ that regulate neurotransmitter function and thus may actually lead to opposite functional effects to those anticipated. For example, DA D2 autoreceptors inhibit DA neuronal firing and DA release from dopaminergic neurons; thus, DA D2 antagonists can increase DA release via presynaptic (autoreceptor) mechanisms (see Ford 2014). Support for Michael Frank’s modelling of D2 receptor actions has depended to some extent on interpreting the effects of haloperidol as working in part at DA D2 autoreceptors (e.g. Frank and O’Reilly 2006). Recently, we were able to report tri-phasic effects of intra-caudate quinpirole on reversal learning in monkeys, with low and high doses impairing performance and intermediate doses significantly improving learning (in terms of errors to criterion) (Horst et al. 2019). We have also observed sometimes ‘paradoxical’ improvements in cognitive performance of highly selective D2/3 receptor antagonists such as sulpiride (Mehta et al. 1999). Later studies by Mehta et al. (2008) showed that the commonly used dose of 400 mg sulpiride occupies only about 30% of striatal D2 receptors. Therefore, in the study of Eisenegger et al. (2014), we obtained ethical permission to administer a dose of 800 mg to healthy volunteers, which occupies about 60% of striatal D2 receptors (and which in fact produced no adverse side effects). We could therefore be fairly sure that any presynaptic effects at the mid-brain level would be overcome by the large D2 striatal receptor blockade (although note there are also striatal D2 terminal autoreceptors that may exert additional effects). We reduced variability when assessing effects of drug dose by capitalising on individual differences in plasma levels of the drug. Moreover, based on previous behavioural findings relating genetic polymorphisms to learning parameters, including reversal (Frank et al. 2007; Jocham et al. 2009; den Ouden et al. 2013), we also stratified the population of human volunteers by recruiting individuals with and without the DA D2 receptor Taq1A polymorphism, as the minor A1 allele has been associated with a reduction of up to 30% of striatal DA D2 receptor density (Thompson et al. 1997). This stratification also potentially helped to dissect possible D2 versus D3 receptor influences.

Sulpiride had very clear-cut effects that were similar in several ways to those of haloperidol in the study by Pessiglione et al. (2006). There were effects on appetitive but not aversive learning, possibly consistent with Schultz’s findings in monkeys (Schultz et al. 1997). Notably, the drug impaired performance, but its effects mainly appeared to be at asymptotic levels of discrimination rather than in its initial acquisition. In fact, this is again similar to the effects reported by Pessiglione et al. Whilst the effects in the entire sample of volunteers were statistically marginal, the findings did reach significance when the influence of dose was introduced (low versus high plasma levels of sulpiride) and there was also a genetic influence whereby the volunteers with the A1 polymorphism exhibited the largest effects.

**Fig. 2:**
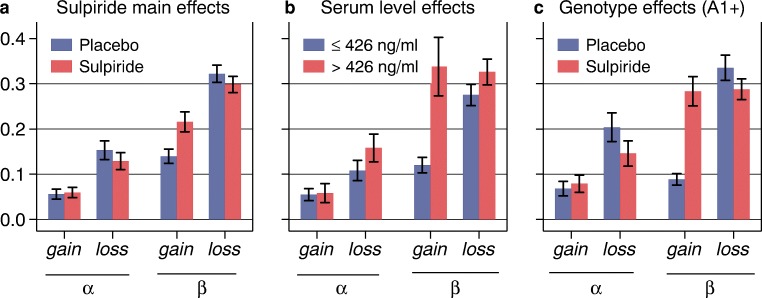
Effects of sulpiride on reinforcement learning model parameters in human volunteers. Effects were restricted to ‘temperature’ (*β*) rather than learning rate (*α*), were for gains only, and were exaggerated by higher plasma levels of sulpiride and in participants with the A1+ genotype of the Taq1A polymorphism for DA D2 receptors. Parameter estimates of the Q-learning model were derived across drug, serum value and genotype groups, separately for the gain and loss domain. **a** The temperature parameter *β*_gain_ was significantly higher in the sulpiride group (57% increase compared to the placebo, *P* = 0.005), but the learning rate *α*_gain_ was not affected, and there were no effects in the loss domain (*α*_loss_, *β*_loss_). **b** Higher sulpiride serum values selectively affected the temperature parameter gain (183% increase in high compared to low serum values, *P* = 0.001), with no effects on either *α*_gain_, *α*_loss_ or β_loss_. **c** Pronounced sulpiride effects on *β*_gain_ were observed in A1+ genotype carriers (211% increase following sulpiride compared to placebo administration, *P* < 0.001), but not in A1–genotype carriers. Reproduced from Eisenegger et al. (2014) with permission of the publishers

All of these findings were further confirmed by the use of a ‘softmax’ Q-learning algorithm similar to that used by Pessiglione et al. (2006) (Box 1), which showed that the effects were confined largely to the temperature parameter *β* (Fig. 2). This precision achieved by the computational model of the drug effects is impressive and has exciting implications, although we are left to interpret what the effects on temperature (*β*) mean. One possibility is that the parameter reflects ‘exploration’ (versus ‘exploitation’) of the reinforcement contingencies (Cohen et al. 2007). From a computational perspective, exploration versus exploitation has been related to changes in tonic DA function in the basal ganglia (Humphries et al. 2012) and from genetic evidence in humans, exploration has been shown to be affected by polymorphisms of the enzyme catechol-*O*-methyl transferase, which regulates DA in the human prefrontal cortex (Frank et al. 2009).

The lack of effect on learning rate is perhaps surprising in view of the presumed role of dopamine in learning. However, it should not be forgotten that D2 receptors have been linked with tonic rather than phasic modes of dopamine function and that the prediction errors are supposed to be a function of the latter (Dreyer et al. 2010). One hypothesis that emerges from the findings is that the D1 DA receptor may be more directly implicated in learning rate (parameter *α*). Unfortunately, D1 DA receptor antagonists have not been widely used in human psychopharmacology to date due to the difficulty in obtaining them for studies in healthy volunteers. However, there is a clear prediction that D1 agents would affect *α*. Such a result would also validate the sensitivity of the probabilistic learning paradigm employed by Pessiglione et al. (2006) and Eisenegger et al. (2014), which may possibly be more sensitive to effects on *β* than *α*.

### Computational modelling applied to a dopaminergic animal model of schizophrenia

A second example of the use of computational approaches to understanding effects on learning of dopamine manipulation comes from an attempt to determine the functional sequelae of DA depletion from the prefrontal cortex in marmoset monkeys (Clarke et al. 2014) to model some of the presumed changes occurring in schizophrenia, which have been proposed to reflect a reciprocal balance between cortical DA underactivity and subcortical DA over-activity (Weinberger 1987; Meyer-Lindenberg et al. 2002). This focuses attention on prefrontal cortical D1 receptors which have been reported to be upregulated in unmedicated schizophrenia and may reflect a compensatory response to disorder-related down-regulation of prefrontal DA (Abi-Dargham et al. 2012).

The experimental depletion in marmosets was effected by 6-hydroxydopamine and restricted to the orbitofrontal cortex. Studies using ligand-based positron emission tomography (PET) showed that this treatment caused a reduction of striatal D2 receptor availability consistent with a striatal upregulation of DA function. The monkeys were tested behaviourally using a range of instrumental probabilistic visual discrimination tasks, structurally similar to that employed for the human studies described above. They were trained to respond to two multi-coloured abstract shapes presented on a touch-sensitive screen. One of these stimuli was probabilistically rewarded with fruit juice, e.g. 80% of trials, whereas the other was only rewarded 20% of the time. Negative feedback (20% and 80%) was provided by aversive white noise. Thus the reinforcement contingencies were quite similar to those employed by Eisenegger et al. (2014) and Pessiglione et al. (2006), above. The optimal strategy in such a discrimination is of course to respond exclusively to the ‘majority’ reinforced stimulus, i.e. 80%. This does mean that the marmoset has to remember the overall associative strengths associated with its choices and ignore, for example, occasional punishment for responding to the 80% stimulus, and occasional reward associated with the ‘minority’ (20%) stimulus. Disproportionate reactivity to such immediate feedback, e.g. to shift away from the majority stimulus following ‘spurious’ punishment, or to stay with the minority stimulus following ‘spurious’ reward, would both serve to retard overall learning. Thus, the reinforcement contingencies in this task can be described as occasionally being ‘misleading’ and affecting their overall ‘truthfulness’ or ‘falsity’, i.e. ‘veracity’, with respect to the overall discrimination task.

Bearing in mind these subtle contingencies, the behavioural changes caused by OFC DA depletion were quite complex and initially difficult to characterise. The OFC DA depleted monkeys were faster to learn, i.e. they made fewer errors to criterion than sham-operated controls. More detailed analysis revealed that this was because the OFC DA depleted monkeys were less affected by ‘false’ punishment, so that they more rapidly discriminated the 80% reinforced, majority stimulus with fewer shifts to the 20% reinforced, minority stimulus. The OFC DA depleted monkeys were not though less sensitive to ‘true’ punishment. Deeper analysis however revealed some difficulties with this simple formulation. Although the effect clearly depends on the prior history of outcomes, when behaviour was analysed for dependencies further back in the sequence of trials (n-back), also taking into account apparent stimulus preferences of the monkeys, this failed to provide a satisfactory description of their behavioural choices.

Therefore, in order to gain further understanding of this change in behaviour, it was characterised further using computational reinforcement learning models. Two major classes were considered. The first was termed ‘model-based’ (or ‘declarative’) learning, in which the subjects are assumed to have expectancies about outcomes and track the reinforcement probabilities with varying degrees of certainty, based on incorporating the current evidence into their prior estimates of them. Thus an ‘ideal subject’ would estimate the overall probability of reinforcement for each stimulus (utilising logical inference to update the associative strengths or values of both stimuli on each trial rather than simply focusing on one stimulus), represent its uncertainty about those estimates, and choose so as to maximise the reward obtained. The subject might in principle have elaborate hypotheses about the structure of the task, for example, anticipating when reversals may occur on the basis of sequences of trial outcomes. This is essentially an ‘optimal Bayesian’ class of models.

The second class of models was described as being ‘model-free’ (the animal does not form a ‘model’ or declarative set of expectations about the environment) and depends on simple reinforcement rules. Value-based delta reinforcement learning algorithms simply assign a value to each stimulus or action in a single update manner, and choose accordingly; the values are updated according to rules and parameters determining the impact of reward or punishment (e.g. sensitivity to reward or punishment, or to reinforcement in general). There is thus no overall strategy for representing the environment as a whole. Some additional parameters were also defined. One of these was the tendency to prefer one stimulus over the other, irrespective of its reinforcement contingencies—i.e. the likelihood of repeating a choice of that stimulus, or ‘stimulus stickiness’. The second was an analogous tendency to prefer one response location because it had been chosen on the previous trial (‘side stickiness’). Consideration of these local strategic tendencies appeared likely to explain some of the behavioural choices of the marmoset, as had earlier been acknowledged by the type of analysis advocated by Sahgal and Clincke (1985) described above. Full computational details of these two classes of model can be found in Clarke et al. (2014).

Thus, several different models were formulated for the behavioural data and tested against one another for the best fit to the data using a standard method called the Bayesian Information Criterion (BIC), which takes into account such factors as the number of parameters employed (fewer being better). (Statistical methods move on, and more recent work has improved the optimal method for Bayesian model comparison; see Gronau et al. 2017.) The ‘winning’, simple reinforcement learning model (Box 1) included parameters for (1) sensitivity to overall reinforcement, regardless of valence, (2) stimulus stickiness and (3) side stickiness. OFC DA depletion modulated the model’s parameters, affecting both reinforcement sensitivity and local response strategies. Specifically, it reduced side stickiness and increased reinforcement sensitivity (irrespective of whether for reward [gain] or punishment [loss]). Of these two effects, the latter was most important for simulating the observed reduction of errors to criterion and was also found to correlate negatively with D2 receptor binding in the head of the caudate nucleus (but, importantly, not in the OFC itself). This reduction in errors could be inferred to correlate with increased DA levels in the head of the caudate. By contrast, the side stickiness parameter correlated better with D2 receptor binding in the body of the caudate, which is consistent with the role of the dorsal striatum in egocentric spatial processing (e.g. Brasted et al. 1997).

These results lead to several interesting implications. First, they are consistent with a view that striatal DA changes are not only important for reinforcement learning, but additionally may affect local strategies such as side bias, probably via effects on striatal tonic levels (as the OFC DA depletion did not affect phasic striatal DA release, determined by microdialysis of this region with K^+^ pulses). The reduced side bias may even reflect an increased tendency to ‘exploration’ (c.f. Cohen et al. 2007), analogous to effects of systemic d-amphetamine to increase response switching in an uncertain environment (Evenden and Robbins 1983) and also to the strategies of acute psychotic patients exposed to a random 2-choice guessing game (Frith and Done 1983). Indeed, other studies of patients with schizophrenia using a similar probabilistic reversal learning procedure (e.g. Waltz and Gold 2007; Waltz et al. 2007, 2013; Schlagenhauf et al. 2014) have also shown changes in parameters of reinforcement learning, illustrating the translational potential of this approach. Specifically, medicated first episode patients with schizophrenia exhibit probabilistic reversal learning as an apparent consequence of increased switching and impaired reinforcement sensitivity (Waltz et al. 2013). Whilst D2 receptor antagonists certainly affect probabilistic reinforcement learning parameters in healthy volunteers, as we have described above, the study by Schlagenhauf et al. 2014) confirmed that the behavioural deficits in probabilistic reversal were also present in unmedicated patients with schizophrenia. The latter study also showed that it was feasible to use computational modelling to identify individual differences in how patients may approach tests such as probabilistic reversal and thus provide a basis for stratification that may help to resolve heterogeneity in clinical populations.

### Computational modelling applied to a serotoninergic translational model of affective disorder

The probabilistic (reversal) learning paradigm has also been used in the context of defining certain deficits in depression. Apart from the relevance of changes in reinforcement learning in this patient group, some early studies noted a particularly relevant effect that may relate directly to certain symptoms in depression associated with Beck’s ‘negative set’—the tendency to focus on negative aspects of experience. Thus, Murphy et al. (2003) reported that patients with major depressive disorder exhibited a significant tendency to shift responding after negative feedback. Whilst this may appear to be a normal consequence of Thorndike’s (1898) Law of Effect (see Thorndike 1911), it is clearly disadvantageous in certain circumstances when, as in the 80:20 reinforcement contingency for the ‘majority’ stimulus, it is appropriate to stay after a loss or negative feedback rather than to shift. This aberrant shifting behaviour is obviously disadvantageous and was later shown to occur in unmedicated patients with depression in an fMRI setting, where it was correlated with the absence of a deactivation in the amygdala that usually occurs in control subjects in this task (and also with a possibly regulatory absence of a prefrontal cortical activation that hypothetically regulates this amygdaloid response) (Taylor Tavares et al. 2008).

Other studies have focused on possible neurochemical correlates of this apparently heightened reactivity to negative feedback, specifically mediated by serotonin—guided by the fact that depression often responds to selective serotonin reuptake inhibitors (SSRIs) (Anderson et al. 2008). These effects of medication are a major source of evidence in support of the serotoninergic hypothesis of depression, which has some additional limited support from PET studies of the status of 5-HT receptors and the 5-HT transporter in major depressive disorder (e.g. Reimold et al. 2008; Kaufman et al. 2015).

Chamberlain et al. (2006) reported that low doses of the SSRI citalopram impaired performance on a probabilistic learning paradigm in healthy volunteers. Skandali et al. (2018) recently followed up these findings in a similar group of volunteers to show increased shifting in response to negative feedback.

In order to test these effects more mechanistically in a rodent model, Bari et al. (2010) designed a spatial version of the probabilistic reversal task in which reinforcement was assigned according to 80:20 and 20:80 contingencies. Dose-related effects of both acute and sub-chronic citalopram were investigated. Effects of central depletion of 5-HT were also determined following intra-ventricular application of 5,7-dihydroxytryptamine, leading to profound forebrain 5-HT loss. The main findings are summarised in Table 1. As can be seen, the effects of an acute low dose (1 mg/kg) and high (10 mg/kg) dose were almost opposite in direction. Importantly, the acute low dose simulated what has been shown human studies, i.e. increased lose-shift behaviour that interferes with the number of reversals achieved, whereas the acute high dose (which presumably produces a net increase in 5-HT transmission via its effects on the 5-HT transporter) improved overall performance. Interestingly, repeated or sub-chronic citalopram also improved reversal performance although via increased win-stay rather than reduced lose-shift behaviour, suggestive of some other neurochemical action. Finally, profound depletion of forebrain 5-HT produced a pattern of findings that resembled acute low-dose citalopram. Overall, these findings are consistent with the human findings, but they do not identify the neural loci at which these effects occur.

**Table 1:**
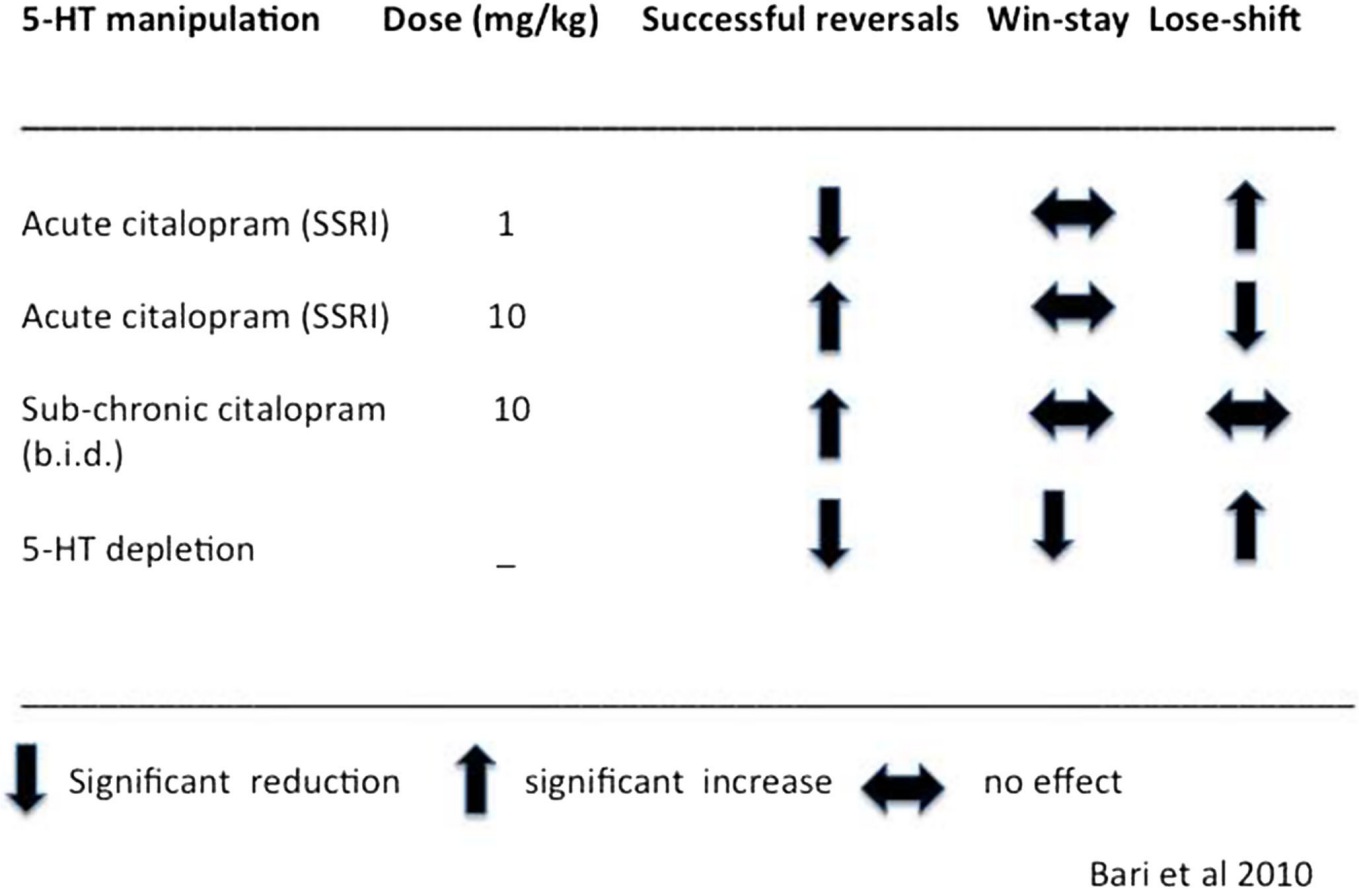
Effects of 5-HT manipulations on probabilistic spatial reversal in rats

Functional brain imaging studies of the probabilistic learning and reversal task have implicated fronto-striatal systems as well as the amygdala (Cools et al. 2002; Pessiglione et al. 2006; Taylor Tavares et al. 2008). Therefore, in a study by Rygula et al. (2014), we focused on effects of regional 5-HT depletion (via 5,7-dihydroxytrypamine) of the amygdala and orbitofrontal cortex in marmosets, using a form of the visual probabilistic reversal task employed by Clarke et al. (2014) for studying effects of orbitofrontal DA depletion.

**Fig. 3:**
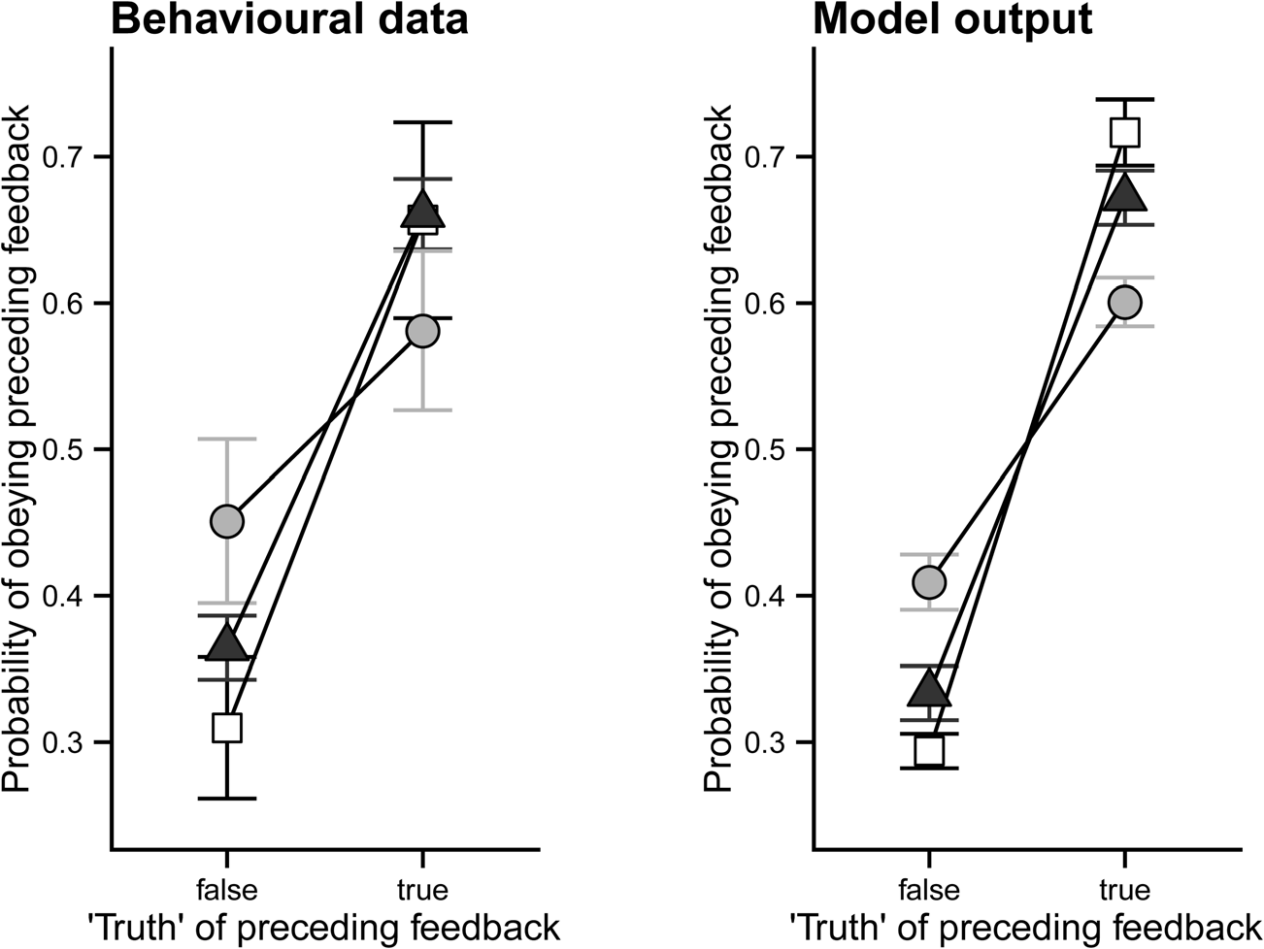
Retrodicting actual behavioural data in probabilistic learning by marmoset monkeys by the ‘winning’ (best-fitting) reinforcement learning model (right). Open squares, sham-operated group. Filled circles, amygdala 5-HT depleted group. Filled triangles, orbitofrontal 5-HT depleted group. The amygdala 5-HT depleted group exhibited impairments in responding to both ‘misleading’ and ‘truthful’ feedback, which were accurately modelled as a deficit in reinforcement sensitivity. Reproduced from Rygula et al. (2014)

These treatments, which produced up to 80% local depletion of 5-HT within about a month after surgery, had behavioural effects analogous to those seen in the human studies. 5-HT depletion in the amygdala impaired performance on the visual probabilistic reversal by increasing the effectiveness of ‘misleading’ negative feedback. OFC 5-HT depletion had similar (but only trend level) effects. However, computational modelling during the learning phase found, perhaps surprisingly, that the behavioural data for that stage in both sites could be accounted for with a relatively simple delta rule reinforcement learning model, similar to that used in Clarke et al. (2014) for the orbitofrontal DA depletion experiment described above. The effects on probabilistic learning could mainly be explained by reductions in reinforcement sensitivity (operating similarly for reward and punishment), both for 5-HT depletion of the amygdala and for the weaker effects observed for the orbitofrontal cortex. However, increased ‘stimulus stickiness’ (i.e. the tendency to repeat responding to the same visual stimulus, regardless of feedback; Box 1) also offered a potential explanation of some of the data at the latter site, whereas the amygdala 5-HT depletion tended to have opposite effects on stimulus stickiness. These models were generated and tested in the same way as before; the final test was to determine to what extent they could simulate the original behavioural data (Fig. 3).

In this example, computational modelling has not fully vindicated the analysis that manipulation of 5-HT function specifically enhances effect of misleading negative feedback in favour of a more general detrimental action on reinforcement. This needs to be resolved by further experiments and analysis. It was not feasible to apply the computational model to the reversal phases in Rygula et al. (2014) because of the relative lack of data and the well-known problem of ‘over-fitting’ the model. The modelling does raise the possibility that not only is the impact of false negative feedback affected by 5-HT, but also that of false positive feedback—in other words, is the tendency to repeat rewarded responses for the minority stimulus also increased? This parameter has not previously been analysed, e.g. in the study of Bari et al. (2010) or the clinical studies, generally because these events did not occur at sufficient frequency.

On the other hand, the effect of orbitofrontal 5-HT depletion may be compatible with a number of other findings. Seymour et al. (2012) found that acute dietary tryptophan depletion in healthy human volunteers tended to increase stimulus stickiness, and the findings may also contribute to our understanding of the effects of orbitofrontal 5-HT loss on deterministic visual reversal learning in marmosets (Clarke et al. 2004). A parallel modelling project has been analysing the performance of patients with obsessive-compulsive disorder (OCD) and stimulant drug dependence on the visual probabilistic reversal learning task, finding opposite effects, for example, on stimulus stickiness (greater in stimulant dependence, but reduced in OCD) (Kanen et al. 2019). These examples provide considerable hope that it may ultimately be feasible to generalise findings of effects of neurotransmitter manipulations from experimental animals (rodent and monkeys) to humans, including patients, and the effects of medications in a new translational agenda that utilises computational modelling as a unifying strategy for reducing differences among experimental test paradigms and allowing generalisations at a more theoretical level.

### Model-based versus model-free learning

Most of the modelling we have performed thus far on data from experimental animals has found that rather simple, reinforcement-based models have proven adequate to account for the data rather than the more representationally elaborate ‘model-based’ learning now increasingly used to characterise human performance. The two-stage Markov decision-making task introduced by Daw (2011) enables a direct comparison to be made of effects of drugs or neurochemical manipulations on model-based versus model-free learning, in the same individual. A full description of the task and its rationale is provided elsewhere (Daw 2011). In general, it has some affinities with the simpler probabilistic learning in that it is sometimes optimal to resist the usual urges to repeat successful choices and to shift from unsuccessful ones, in order to maximise reinforcement. This task has increasingly been employed to make comparisons between patient groups and healthy controls, commonly finding that there is a shift towards model-free learning, for example in stimulant dependence or OCD (e.g. Voon et al. 2015). Pharmacological studies are at present at an earlier stage, however. Wunderlich et al. (2012) reported, perhaps surprisingly, that a dose of L-DOPA selectively enhanced model-based learning, perhaps as a consequence of its actions in the ventral striatum (Deserno et al. 2015). Had it been ethically permissible to employ higher doses, these may have alternatively had effects on model-free behaviour via the dorsal striatum. Worbe et al. (2016) modified the task to include a parallel set of contingencies to avoid loss (in addition to the usual ones for gain) and studied the effects of acute dietary tryptophan depletion. This treatment had striking effects on model-based versus model-free learning that depended on affective valence. Tryptophan depletion enhanced model-free processing when it was appetitive, but model-based performance when aversive. This strongly suggests again that a manipulation of 5-HT can have asymmetrical effects on reward and aversive processing, as suggested by the original observations on patients with depression (Murphy et al. 2003) and some of the pharmacological studies of 5-HT function. We can anticipate further studies with these evolving test procedures to be made with more precise neurobiological interventions in experimental animals.

## Summary and conclusions

‘Computational psychopharmacology’, like computational psychiatry (Heinz 2017), is a young and nascent field that seems to be worthy of further development, especially in a translational context that links the two. We have summarised recent findings from a specific set of test paradigms based around probabilistic learning and reversal learning that show some promise in uncovering new principles about drug or neurochemical effects, of possible relevance to pharmacotherapeutics. However, rather than merely describing behavioural effects of drugs, the emergent models will also have to make contact with plausible psychological constructs, explain psychiatric symptoms, and make testable predictions for other test settings and situations, in order for them to become fully pragmatic and paradigmatic.
